# A Systematic Study Site Selection Protocol to Determine Environmental Flows in the Headwater Catchments of the Vhembe Biosphere Reserve

**DOI:** 10.3390/ijerph19106259

**Published:** 2022-05-21

**Authors:** Anesu D. Gumbo, Evison Kapangaziwiri, Fhumulani I. Mathivha

**Affiliations:** 1 Department of Geography and Environmental Sciences, University of Venda, Thohoyandou 0950, South Africa; 2 Hydrosciences Research Group, Council for Scientific and Industrial Research (CSIR), Pretoria 0001, South Africa; ekapangaziwiri@csir.co.za; 3 Department of Hydrology, University of Zululand, Empangeni 3886, South Africa; mathivhaf@unizulu.ac.za

**Keywords:** climate change, case study, environmental assessment, data scarcity, science communication, selection protocol

## Abstract

Developing nations will be worst hit by the impacts of climate change because limited resources hinder the spatial reach of climate studies, effort, and subsequent implementation to help with the improvement of livelihoods. Therefore, finding the best-case study is an essential undertaking in environmental assessments. This study explains one systematic approach to selecting a study site for an environmental assessment project. A desktop review of relevant literature, a simple factor scoring assessment process, reliance on expert opinion, and a field survey for ground-truthing were conducted. The desktop review showed the most critical factors to site selection. The scoring of these factors selected those that were crucial for the study. Experts validated the results and suggested the best study site among the ones identified. While the design is simplified, the proposed approach selects the most appropriate study site for environmental assessments.

## 1. Introduction

It is imperative and apparent that understanding changes in climate, and subsequently their implications on society and the environment (especially the devastating adverse effects), is a necessary undertaking. Integrating several disciplines to inform mitigation and adaptation options to the unprecedented climate changes and their devastating impacts has become important [1]. The general scientific fact is that climate change will increase the occurrence, magnitude, and frequency of extreme weather events and potentially lead to loss of food production and livelihoods [2,3]. A crucial global message is that there is a need for robust responses to arrest the adverse impacts to save lives and livelihoods, particularly in the most vulnerable societies of the world [4].

According to [5], vulnerability to climate change is regarded as the degree to which a system is susceptible to and unable to cope with the adverse effects of climate change. How much a system is exposed, its sensitivity to the exposure, and its adaptive capacity provides an understanding of how vulnerable it is. Climate change impacts are distributed unevenly around the globe [6], based on geographical location, level of vulnerability, level of preparedness and access to necessary resources to implement mitigation and adaptation, and societal capacity to understand and perceive climate changes [7]. Area-specific climate change assessments are, thus, vital undertakings to address the global challenge at local levels to increase the adaptation capacity [8]. The global agenda has been placed on climate change mitigation and adaptation. 

Developing countries, however, have low mitigation capacity, and adapting to these changes is ideal and many countries are developing their own National Adaptation Plans (NAPs) [9]. These strategies to adapt to climate change are backed up by science which projects future possible scenarios. Southern Africa is one of the regions to experience some of the most adverse effects of climate change. Studies e.g., [10,11,12,13] indicate that the region’s vulnerability is high, while its preparedness is low to nonexistent. Several issues such as poverty, corruption, political instability, limited access to climate information, pandemics, and other problems influence the responsive capacity of countries in southern Africa.

The past few decades have produced scientific reports on the projected magnitudes and directions of changes in the climate and the potential impacts on water resources’ quality and quantity [14,15,16,17,18,19,20]. Improved projections are due to massive technological advancements [21,22] that have improved data collection and analysis methods and the efficiency and robustness of environmental assessment tools [23]. Such improvements have reduced some uncertainties related to environmental assessments and built more confidence and dependability in climate change projections to influence policy and decision making. High confidence in climate projections has made science a vital pillar to support the development of mitigation and adaptation strategies, approaches, and technologies necessary to combat the potential shocks of a changing climate [24]. These adaptation strategies are tested and modified in areas that have human–environment interactions. Several areas with these characteristics have been demarcated around the world and have been termed biospheres.

Biosphere reserves have been created as study sites to understand the climate change–human–environment interactions. The United Nations Educational, Scientific, and Cultural Organization (UNESCO) regards biosphere reserves as learning places for sustainable development [25]. They are sites for testing interdisciplinary approaches to understanding and managing changes and interactions between social and ecological systems, including conflict prevention and management of biodiversity. Consequently, there are 727 established biosphere reserves in 131 countries [25] whose purpose is to provide a learning environment for sustainable development in diverse and fragile but significant ecological regions. They utilize different socio-economic contexts to enhance the lives and livelihoods of the communities within them [26]. Local solutions to global challenges affecting sustainable development are developed and tested in these reserves. The value of these reserves in mitigating and adapting to climate change is significant, offering opportunities for case studies to be conducted as learning platforms. The Vhembe Biosphere Reserve (VBR) is a protected area in the Limpopo Province of South Africa. Its spatial extent limits the implementation of research work to only selected areas. These selected areas need to be carefully identified as they become the general representation of the biosphere and the results gathered from their study would need to be adopted by similar areas. Out of the 10 biospheres in South Africa, the VBR was selected as the area of study because of a high rural population vulnerable to climate change impacts. It is a data-deprived area that requires focus to generate information/data to aid informed decision making. Data availability and data quality are common problems in many areas in Africa [27] and this affects studies being carried out in such areas. Climate change impacts on water resources are affected by the lack of adequate and good quality time series of hydrometeorological data [28]. Creating protocols for carrying out research in such areas becomes critical to informing sustainable decision making. 

Quantifying water resource availability against climate change is an important undertaking as water is central to sustainable development. Developing nations, such as the southern African regions, are mainly rural [29] whose socio-economic activities are closely tied to the availability of water resources. Many rivers in the southern African region have high flows during the rainy season and low to no flows during the dry season [29]. The dry season is persistent throughout the year and communities rely on the low flows to sustain their socio-economic activities and the environment. The South African Water Act of 1998 identifies the environment as a legitimate and important user of water resources in any sub-basin requiring that a determined amount of water be reserved. Hence, the environmental water requirements (popularly known as e-flows worldwide) are important to catchment water management. In the Water Act of 1998, the e-flows are referred to as the reserve. The quantification of these e-flows in South Africa is an ongoing undertaking of great importance to water resources’ budgeting and accounting [30,31]. The task generally requires a water practitioner or user to provide a holistic assessment utilizing any one of the several acceptable approaches and tools. This is to fully understand the ecological functioning and value chain of a given sub-catchment and determine a sensible and viable e-flow requirements regime for the survival and sustenance of the riverine ecosystem. The choice of a representative area of study or river stretch should then be carefully considered to produce results that can inform decision making for the chosen area and its implementation in other areas of similar characteristics.

Ref. [32] explained that case studies are intense meticulous studies of a particular area to generate results that can be generalized over a more extensive set of units. They are used in exploratory research [33], where no past data or a few referral studies exist. Therefore, identifying a study site that can address one or several objectives of the overall aim is essential. The overall aim of the intended study for which the case study site(s) must be selected is to determine the e-flows for headwater catchments that are suitable and necessary to sustain riverine ecology within major selected parts of the Vhembe biosphere and assess the potential impacts of a changing climate on the availability and sustainability of these flow regimes. Several headwater catchments are potentially available for study within the reserve. However, because of several factors (time, funding, technical expertise, and best representative of areas in the VBR), the study will have to choose one or a few sub-basins to use. Results would then have to be extrapolated to the rest of the headwater catchments. Thus, representative study sites must be selected to generate and establish baseline information and methodologies that could be applied in the other areas. This paper proposes a step-by-step and objective approach to determine the ideal case study site(s) to determine e-flows within the VBR. 

## 2. Study Area

**Figure 1:**
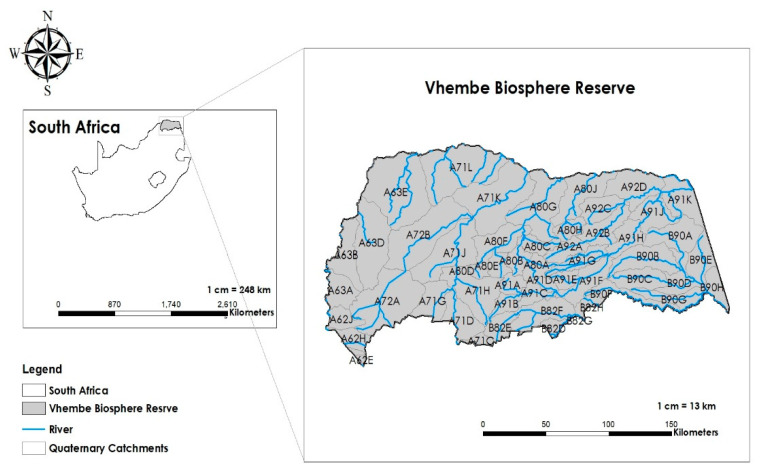
A map showing the spatial extent of the Vhembe Biosphere Reserve (VBR) and the catchments for potential study sites.

The Vhembe District Municipality and the Blouberg Local Municipality (within the Capricorn District Municipality) together form the boundaries of the VBR. Figure 1 shows the location and spatial extent of the VBR. The VBR is the largest of the 10 biosphere reserves in South Africa [34]. It reaches 30,701 km^2^, supporting a human population of approximately 1.5 million, with 97% of the population rural [35]. The Vhembe region holds a unique and extraordinary biological and cultural diversity in the Soutpansberg and Blouberg Mountains, together with the Mapungubwe World Heritage Site and northern Kruger National Park [35]. Local communities have a high, direct reliance on natural resources for livelihood, with 66% of households relying on firewood for fuel and subsistence agriculture [35]. Agriculture and tourism are the major socio-economic activities that sustain livelihoods in the reserve. 

Several rivers within the VBR maintain diverse flora, fauna, and riverine ecosystems. These rivers include the Luvuvhu, Dzindi, Nzhelele, Tshipise, and several others, either tributary or pour directly into the Limpopo River. The transboundary nature of the Limpopo River brings about delicate management issues that need a coordinated effort for all riparian countries to benefit from the river’s ecosystem values and functions. The VBR comprises three biomes: the Savannah, Grassland, and the Forest [35]. This implies the existence of rich biodiversity, some of which is endemic to the area. Such biodiversity requires coordinated and well-structured management strategies to ensure conservation viability. The general growth in population and dependence on the environment for energy and livelihood negatively impacts natural resources, and a changing climate is likely only to aggravate the situation.

## 3. Materials and Methods

The study used several methods to determine which area(s) would be ideal for determining appropriate e-flows within the headwater catchments of the VBR. The study is based on a systematic review of relevant literature, a simple factor scoring assessment process, experts’ opinions, and a field survey for ground-truthing. 

### 3.1. Systematic Review

A detailed desktop scan of literature was carried out to determine the study sites used, especially the motivation for selecting the study sites. Literature on or referring to the VBR was reviewed to understand the previously carried out studies’ nature and thrust. This also determined the potential gaps in scientific knowledge and understanding that require additional studies to address them successfully. As information is made available through research, these gaps are plugged, promoting sustainable management for this fragile ecoregion. Google Scholar identified journal articles using the keywords *‘study site selection for environmental assessment’* and about 78,300 articles. A 10-year coverage of literature (2011–2021) was adopted for the study. Selection and rejection criteria (Table 1) were used to select the ideal research for review. The review only analyzed peer-reviewed work and excluded grey literature. Using the selection criteria, seven peer-reviewed journals were critically reviewed. A thematic analysis of the selected articles determined the significant factors that contributed to selecting the study site. The complete review of the selected literature led the study to adopt an exploratory factor analysis (EFA) to determine (without explicit ranking) the factors influential in selecting a study site.

### 3.2. Exploratory Factor Analysis

Several factors influencing the selection of a study site were obtained from the desktop review and were used to inform the selection criteria for this study. These included accessibility, availability of funds, familiarity with the area, and research gap. The exploratory factor analysis (EFA) was implemented to reduce the identified factors to only those regarded as crucial to achieving the aim and objectives of the study. A factor-scoring criterion was developed to select the variables that could comprehensively address the aim of the study and the associated specific objectives. The variables were scored in importance from very important to not important.

### 3.3. Expert Opinions 

Valuable expert opinion was solicited from knowledgeable practitioners with extensive work experience within the VBR. The definition of an expert, in this case, is rather loose and much more encompassing than the normal dictionary one. Experts included academics, researchers working or who had worked in the area and were assumed to possess valuable in-depth knowledge of the area’s processes, functions, and importance. The definition was extended to include people who have resided in the confines of the biosphere and have a wealth of knowledge accumulated over many years of observation of relevant phenomena in the area and can therefore discuss and share valuable insights on the scientific queries. 

### 3.4. GIS-Based Assessment and Ground-Truthing 

Geographical Information Systems (GIS) were used to show all the potential sites selected to implement the study. This provided visual confirmation and display of the spatial distribution of the selected study sites in a way that could show their advantages over the other competing sites. Google Earth and available land use maps (e.g., the 2018 land use map of SA and its recent update from the Department of Forestry, Fisheries, and the Environment) were used to determine the land uses and land cover distributions, including the location of hydraulic structures, as well as vulnerability to possible degradation around the study site(s), among others. Site visits were then carried out as the last activity to familiarize with the area and confirm assumptions and GIS findings. 

## 4. Results 

### 4.1. Systematic Review Results

The literature review sought to determine what others considered important factors when selecting a study site for their environmental research. Information on where similar studies have been carried out and the gaps in research from such studies were identified. Table 2 shows the main factors informing study site selection, with those with an Asterix (*) being most common. A total of 12 factors were drawn from the literature search. 

### 4.2. Factor Scoring Analysis Results

The factors derived from the desktop analysis of literature were listed and given a score based on how important they were in meeting the aim and objectives of the study. Table 2 shows the 12 factors used for this study and the factor score results. The factors considered very important for the study had the most influence on the site selection process. Of the 12 factors, 8 of them were regarded as significant. Table 3 shows the results of the selection criteria which indicate that the catchments A91A (Luvuvhu), A92C (Mbodi), and A91B (Sterkstroom) satisfy the criterion created. 

### 4.3. Expert Opinion Findings

From the desktop review, three experts were identified, an aquatic ecologist who has worked within the VBR and two hydrologists who have done extensive work in environmental modeling and climate change impact on streamflow availability. These experts gave guidance on the best site for the intended environmental study. Literature review and factor analysis resulted in selecting the headwaters of the Nzhelele and Luvuvhu River catchments as possible study sites as they met most of the criteria developed. These choices were then validated by experts whose opinions favored sites in the Luvuvhu River Catchment (LRC) as the most suitable.

### 4.4. Findings of GIS Assessment and Ground-Truthing 

**Figure 2:**
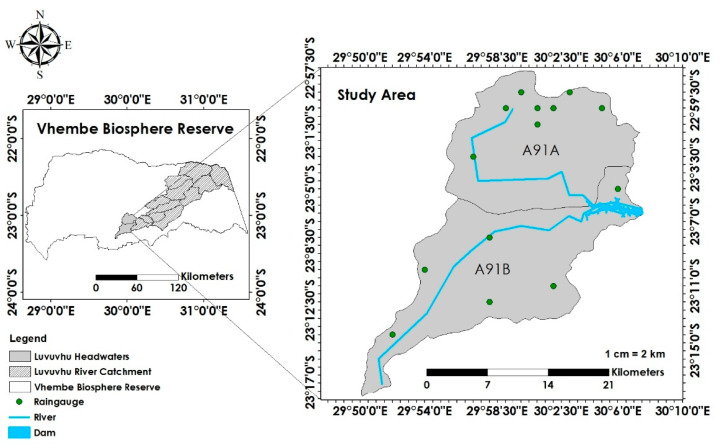
The selected headwater catchments of the Luvuvhu River Catchment in the Vhembe Biosphere Reserve.

The selected quaternary catchments are shown in Figure 2. They are located at the uppermost part of the LRC, making them the headwater catchments for the basin. Several more headwater catchments join the LRC downstream of A91A and A91B. Most of the streamflow drained by the selected headwater catchments collects in the Albasini Dam, creating a different hydrological regime downstream of the dam. It was also confirmed that some parts are densely vegetated and deprived of human interference while others have human activities. The water collected in the dam supports domestic use, agricultural and fishing activities. This phase of the research managed to show the location of the selected study site within the VBR. It confirmed that the objectives of the comprehensive study to be implemented could be achieved. 

## 5. Discussion

The assessment of the literature shed light on the reasons that drive researchers to select study sites. The need to address or understand an environmental problem appeared as the primary reason for site selection. The absence of similar work in an area can prompt a researcher to implement an environmental assessment within a region. This is particularly important where global challenges need to be addressed through sound scientific research [36]. Some regions will be worst affected by climatic changes, such as southern Africa, Asia, and Latin America, mainly due to low mitigation and adaptation capacity [37]. The knowledge of this future possibility requires more scientific inquiry in these regions. The need to contribute locally developed solutions to global challenges encourages researchers to implement case studies, especially in vulnerable regions. The availability of funds through donors, government, or private institutions significantly affects site selection [38]. Funders can influence a study and site selection based on institutional interests and agendas. However, this does not always give the best results in sustainable development due to the often top-down approach. The proximity of the researcher to the study area, and therefore accessibility, also plays a vital role in site selection. This is especially true in academic research as it is often limited in time, funding, and adequate supporting technical expertise. Several environmental assessments are based on the lack of adequate information on a specific environmental phenomenon that influences the direction of research in an area. 

Environmental management and climate change mitigation and adaptation strategies have acknowledged the need for more research to be carried out to aid decision making and policy formulation [39,40]. The study site selection is not well documented, as shown by a lack of publications focused exclusively on this topic in environmental studies. This is not a popular undertaking or conscious decision in environmental studies. However, other disciplines such as health sciences have clear protocols to follow when selecting a study site [41]. They thoroughly analyze the research aim, objectives, and expected deliverables and select the best study area to achieve these. Adopting this approach in environmental assessments will be of great value and could account for common deterrents such as data unavailability. Prior knowledge of what the study requires and targets to achieve against what potential study sites can provide enables the researcher to design the best methodology. It becomes time-consuming and costly when a research design cannot be implemented because the chosen study site lacks the components that enable the methodology to be successfully implemented. This is important in data-scarce regions [42]. Alternative data sources, in such cases, may need to be used, which may require downscaling, interpolations, and extrapolations that the project might not have accounted for in the design phase. This inevitably leads to delays in project implementation and the invasion of additional uncertainties in the generated results, which may reduce the degree of confidence in these results. 

The factor scoring results showed the likely catchments to implement the study. Data scarcity was regarded as a unique factor that, in its availability, would simplify the research and in its absence, presents an opportunity to generate it. Data of sufficient quality and quantity are always required in environmental studies, but often, they are the missing component. Data scarcity is a pandemic in environmental studies and limits research in some areas. However, data-scarce regions are usually the most vulnerable to climate change, and sustainable development of such areas can benefit from local scientific investigations. The selection protocol regarded the lack of observed hydro-climatological data (rainfall, streamflow, evaporation) as the desired factor. These areas are usually understudied because they lack observed historical data [43]. Therefore, it is imperative to carry out scientific investigations in these areas to generate reliable baseline information to support development initiatives. An opportunity arises to carry out a study in a data-scarce area to generate more data for various purposes, including decision and policymaking and creating a methodology that is transferrable to other sites. Designing a methodology that utilizes the bare minimum of data will give a more substantial basis for adoption on other sites. The current study needs to be carried out in headwater regions due to their unique provision of ecological goods and services essential to riverine integrity [44]. 

Due to their hard-to-reach and underdeveloped nature, headwater catchments are subject to limited studies [45]. In the African context, headwaters are data-scarce in terms of observed hydrometeorological data of sufficient quality and literature of previous studies carried on them [29]. Though insufficiently represented in research, these areas are home to a larger rural population that depends on natural resources for its livelihood. Water availability is one natural resource that is important in these communities and its availability is crucial to livelihoods. Southern Africa experiences a long dry period during the year. Low flows are crucial during this period to cater to the socio-economic activities that sustain the riparian rural communities. Understanding the minimum water requirements for the survival of riverine ecosystems is essential to river management, especially in a changing climate. Therefore, the headwater catchments must support rural communities benefiting from them. The study intends to improve rural resilience as the local climate changes. Building resilience through scientific approaches will also rest on the co-production of work to formulate frameworks that can be adopted at the local level using the resources available. The scoring criteria, thus, realized any potential benefits that would accrue to the rural communities in the study area to be of great importance in selecting a suitable study site. 

Ref. [46] discuss the challenges expected in e-flow determination in the river catchment, though no specific e-flows quantities were recommended for the area. They acknowledge that this river is crucial to rural livelihoods as it provides a source of animal protein and ecosystems goods and services to the Kruger National Park downstream of the Luvuvhu River. The importance of the LRC was emphasized as well through the consultations carried out with experts who have worked in the biosphere and e-flow determination. The research utilized the information and recommendations of at least three experts. Literature review and factor scoring managed to further narrow down the site selection. Expert opinion was important to determine which catchments, from the assessments, would best address the overall objectives of the study and provide a good case study. The vast knowledge and understanding of the area, areas that still need researching, knowledge of research that was carried out, and the agreement of the experts led to the selection of the catchments A91A and A91B. According to the experts, the LRC offers an excellent opportunity to study most of the issues that arise in catchment management. The area is rich in biodiversity and carrying out this study will be of great importance to understanding the central role of water in this region. The area currently provides room for collaborative efforts in catchment studies as several projects are currently underway that deal with water and environmental management. Through networking and collaboration, the determination of e-flows in the LRC can be aided by the work being carried such as water quality and distribution of freshwater fish species. The headwaters of LRC have also been demarcated as part of the Strategic Water Source Areas of South Africa that have a unique ability to provide a substantial amount of water resources to the river basin than the other sections of the river [30]. The South African government has embarked on protecting these areas, and studies that demarcate and quantify the water resources potential of these areas are crucial [30]. One expert corroborated this and emphasized the need to carry out the e-flow determination in the LRC and its importance to the national agenda on WSAs. What the research drew from the consultations with experts was that though the criteria are important for determining study sites, input from people familiar with the characteristics of the area is invaluable.

## 6. Conclusions

Understanding the impacts of climate change on water resources and how these influence rural livelihoods is of great value to sustainable development. With the projections that rural populations are more vulnerable to the impacts of climate change, there is a need for science to aid adaptation in these communities. Biospheres provide the opportunity to test the man and environment relationship well. As such, this research sought to determine the best-case study to implement an e-flow determination on headwater catchments within the biosphere. The research, through limitations in funding, time, and human resources formulated a protocol to select the best-case study. Though simple, this case study can be modified accordingly and can be implemented in environmental studies in other areas. The study used a detailed review of relevant literature, a simple factor scoring approach, reliance on expert opinions, and ground-truthing to select the best study site to determine e-flows for the LRC. Several benefits are derived when using a systematic approach to site selection. Environmental research relies on data of sufficient quantity and quality. The availability or lack of this data enables researchers to formulate the most appropriate methodology to implement in their studies. A properly formulated methodology saves time in its implementation. Frequently, environmental research goes beyond the anticipated time as unprecedented hindrances are met during the study. This is particularly true for scholars who usually have limited time, a tight budget, and are on a learning curve. Implementing this systematic site selection approach before they begin their studies will inform them on what is and what is not achievable. At the project level, a proper site selection protocol saves on resources. Southern Africa is limited in its adaptation capacity because of limited resources. Each research carried out in this area should be within a small range on the uncertainty spectrum. Most countries in the region cannot afford to implement research work that drags on for longer than anticipated because of limited resources. The site selection protocol in this study selected headwater catchments that are populated by rural communities and have hydrometeorological data scarcity issues. This was undertaken mainly because most catchments in southern Africa face these challenges as alluded to by [47]. With the technical guidance and the knowledge to generate data, this study is expected to generate new information for the area and the methods used can be transferred to other similar areas in the region. As protocols to study marginalized areas become available, adaptation strategies that cater to all can be achieved. This article emphasizes the importance of carrying out a careful and systematic study site selection as the initial undertaking in any environmental assessment. Such would provide intelligence on the study’s shared challenges and outcomes and, thus, provide a plausible methodology to overcome the challenges and achieve the expected outcomes.

## Figures and Tables

**Table 1:** Literature selection criteria to determine factors that contribute to study site selection.

Search Protocol	Inclusion	Exclusion
Initial Google Scholar search	- English literature.	- Non-English.
	- Peer-reviewed.	- Gray area.
	- Any region.	
Title and abstract review	- Site selection of a study area/case study.	- Sight selection for project implementation, e.g., wind farm, landfill, solar.
	- Climate research.	- Any other study.
Study site description	- Field studies.	- Non-field studies.
	- Areas specific studies.	- Non-area-specific studies.

**Table 2:** Factors influencing the choice of a study site and their level of importance in the intended study. X marks the importance of the factor.

Factor	Importance			
	Very	Just	Neutral	None
Accessibility: ease of reach (near to the researcher). *	X			
Need for human presence at the study site or beneficiation from the headwater catchment/s.	X			
The presence of environmental phenomena to be quantified and understood. *	X			
The rich biodiversity in the area.	X			
Availability of hydrometeorological data of adequate quality and quantity. (*Headwater catchments with missing or no observed time series were more critical than those with).*			X	
The economic value of the area.	X			
Need to test a model or equipment (experimentation).			X	
Past similar work has been carried out in the area. *	X			
Availability of relevant literature.			X	
Influence of expert opinion.	X			
Communication barriers (with stakeholders).				X
Availability of (adequate) funding. *	X			

* Most typical reasons why researchers select a study site.

**Table 3:** The factors that are important to site selection and the possible catchments of study. The tick (✓) shows the quaternary catchments that meet the desired factor and could be chosen for study.

Quaternary Catchment Name.	Accessibility: Ease of Reach (Near to the Researcher).	Need for Human Presence at the Study Site or Beneficiation from the Headwater Catchment/s.	The Presence of Environmental Phenomena to Be Quantified and Understood.	The Rich Biodiversity in the Area.	The Economic Value of the Area.	There Is No Similar Work Done in the Area.	Availability of Experts for the Area.	Availability of (Adequate) Funding.	No Streamflow Data Is Available.
A91A	✓	✓	✓	✓	✓	✓	✓	✓	✓
A91B	✓	✓	✓	✓	✓	✓	✓	✓	✓
A91D		✓	✓			✓	✓	✓	
A91G		✓	✓			✓	✓	✓	✓
A92A		✓	✓			✓	✓	✓	✓
A92C	✓	✓	✓	✓	✓	✓	✓	✓	✓

## Data Availability

Not applicable.
